# Exploring longitudinal patterns of absences, exclusions and attainment in secondary schools in Scotland

**DOI:** 10.23889/ijpds.v9i5.2824

**Published:** 2024-09-18

**Authors:** Morag Treanor, Patricio Troncoso, Lee Williamson, Cecilia Macintyre

**Affiliations:** 1 University of Glasgow; 2 Scottish Centre for Administrative Data Research (SCADR); 3 University of Edinburgh; 4 Scottish Government

## Objective and Approach

We seek to understand the impacts that absences and exclusions have on educational outcomes in secondary school pupils in Scotland. We adopt a Multilevel Structural Equations Modelling approach to analyse data from the newly linked administrative database created under the “Children’s Lives and Outcomes” research strand of the Scottish Centre for Administrative Data Research (SCADR), which includes data from Education Analytical Services (Scottish Government) and Public Health Scotland (NHS) from the period between 2007-2019, and the 2001 and 2011 Census.

## Results

While exclusions, absences and attainment are known to be associated with each other and with individual and structural factors, less is known about how these phenomena play out over time in the Scottish education system. Our analyses focus on a particular cohort of students followed up over their entire schooling period to understand how longitudinal patterns of school absences and exclusions, and other relevant individual and structural factors are interrelated over time. We show that variation across schools and local authorities in these outcomes is significant and substantial in Scotland. We also analyse the impact of these longitudinal patterns on attainment, with an emphasis on mental health wellbeing and familial socioeconomic circumstances.

## Conclusions and Implications

By elucidating further these relationships and interrelationships, we expect our findings to be pertinent to policymakers and practitioners, contributing to inform strategies, policies and/or interventions to reduce the inequalities in exclusions, absences and attainment, ultimately helping to improve children and young people’s school experiences and outcomes.

